# 肠镜下分级对非血缘脐血移植后恶性血液病患者肠道急性移植物抗宿主病的预后评估价值

**DOI:** 10.3760/cma.j.cn121090-20231206-00293

**Published:** 2024-05

**Authors:** 森林 汪, 光宇 孙, 小玉 朱, 雪梅 徐, 飞 叶, 世兰 李, 思 陈

**Affiliations:** 1 中国科学技术大学附属第一医院（安徽省立医院）消化内科，合肥 230001 Department of Gastroenterology, The First Affiliated Hospital of the University of Science and Technology of China（Anhui Provincial Hospital）, Hefei 230001, China; 2 中国科学技术大学附属第一医院（安徽省立医院）血液内科，合肥 230001 Department of Haematology, The First Affiliated Hospital of the University of Science and Technology of China（Anhui Provincial Hospital）, Hefei 230001, China; 3 中国科学技术大学附属第一医院（安徽省立医院）临床病理中心，合肥 230001 Clinical Pathology Centre, The First Affiliated Hospital of the University of Science and Technology of China（Anhui Provincial Hospital）, Hefei 230001, China

**Keywords:** 脐血干细胞移植, 移植物抗宿主病, 结肠镜检查, Cord blood stem cell transplantation, Graft vs host disease, Colonoscopy

## Abstract

**目的:**

探讨肠镜下分级对非血缘脐血移植（UCBT）后出现肠道急性移植物抗宿主病（IT-aGVHD）的恶性血液病患者的预后评估价值。

**方法:**

收集2016年6月至2023年6月在安徽省立医院进行UCBT后出现激素耐药的IT-aGVHD患者50例，比较肠镜下黏膜损伤较轻组（27例，肠镜下分级为Ⅰ、Ⅱ级）和较重组（23例，肠镜下分级为Ⅲ、Ⅳ级）患者IT-aGVHD治疗的有效率、生存率等，回顾性分析影响患者预后的因素。

**结果:**

轻症组、重症组患者在肠镜检查后28 d有效率分别为92.6％和47.8％（*P*<0.001），56 d有效率分别为81.5％和39.1％（*P*＝0.002），最优有效率分别为92.6％和65.2％（*P*＝0.040），差异均有统计学意义。多因素分析发现，肠镜下分级是影响IT-aGVHD治疗有效率的独立危险因素。轻症组、重症组患者肠镜检查后2年的总生存率分别为70.4％（95％*CI* 52.0％～88.8％）和34.8％（95％*CI* 14.8％～54.8％），差异有统计学意义（*P*＝0.003）。多因素分析显示，肠镜下分级、巨细胞病毒感染状态、二线治疗方案及患者的年龄是影响生存的独立危险因素。

**结论:**

肠镜下黏膜损伤程度较轻组（Ⅰ、Ⅱ级）患者的治疗有效率和预后优于损伤程度较重组（Ⅲ、Ⅳ级）。

## Abstract (en)

**Objective:**

To investigate the prognostic value of enteroscopic grading for the prognostic assessment of patients with malignant hematological diseases who developed intestinal acute graft-versus-host disease（IT-aGVHD）after unrelated cord blood transplantation（UCBT）.

**Methods:**

Fifty patients with IT-aGVHD who developed hormone resistance after UCBT from June 2016 to June 2023 at Anhui Provincial Hospital were collected to compare the effective and survival rates of IT-aGVHD treatment in the group with milder enteroscopic mucosal injury（27 cases, enteroscopic grading of Ⅰ and Ⅱ）and the group with more severe injury（23 cases, enteroscopic grading of Ⅲ and Ⅳ）and to retrospectively analyze the factors affecting patients' prognosis.

**Results:**

Patients in the mild and severe groups had an effective rate of 92.6％ and 47.8％ at 28 days after colonoscopy（*P*<0.001）, 81.5％ and 39.1％ at 56 days after colonoscopy（*P*＝0.002）, with optimal effective rate of 92.6％ and 65.2％（*P*＝0.040）, respectively, and the differences were statistically significant. The multifactorial analysis found that enteroscopic grading was an independent risk factor affecting the effective rate of IT-aGVHD treatment. The overall survival rate at 2 years after colonoscopy was 70.4％（95％*CI* 52.0％–88.8％）and 34.8％（95％*CI* 14.8％–54.8％）for patients in the mild and severe groups, respectively, and the difference was statistically significant（*P*＝0.003）. Multifactorial analysis revealed that enteroscopic grading, cytomegalovirus infection status, second-line treatment regimen, and patients' age were independent risk factors for survival.

**Conclusion:**

The treatment efficacy and prognosis of patients in the group with less severe enteroscopic injury（grades Ⅰ and Ⅱ）were better than those in the group with more severe injury（grades Ⅲ and Ⅳ）.

异基因造血干细胞移植（allo-HSCT）是治疗血液系统恶性疾病的重要手段[1]–[3]，常见的移植类型包括亲缘相合、非亲缘相合、单倍体及非血缘脐血造血干细胞移植（UCBT）等。UCBT因移植物可快速获得、人类白细胞抗原相合度要求较低及慢性GVHD（cGVHD）发生率低等优点，成为目前重要的造血干细胞移植类型之一[4]。急性GVHD（aGVHD）是allo-HSCT后常见的并发症，主要累及的脏器包括皮肤、肝脏及消化道。即使经过积极预防，仍有30％～50％的移植后患者会发生aGVHD，且14％的患者为重度aGVHD[5]。肠道aGVHD（intestinal tract aGVHD，IT-aGVHD）是治疗反应差、致死率高的aGVHD类型之一[6]–[7]，IT-aGVHD主要表现为腹泻及腹痛，严重者可出现肠梗阻及便血。重度IT-aGVHD患者多因治疗有效率低、易合并严重的营养不良及感染等因素死亡[8]–[10]。目前，诊断及评估aGVHD严重程度主要依靠患者的临床表现，但对于治疗反应差、存在其他并发症的患者，消化内镜检查可直接观察肠道黏膜的受损程度并能进行肠道组织活检，是IT-aGVHD患者重要的辅助诊疗手段。

既往研究显示，肠道组织病理学检查是诊断IT-aGVHD及评估IT-aGVHD患者预后的重要手段[7]–[8]；但因aGVHD多发生在allo-HSCT早期，此时患者的造血功能尚未恢复，部分患者因血小板输注无效、组织活检出血风险高等原因无法进行肠道组织病理学检查。消化内镜可通过肠镜下黏膜表现对肠道黏膜的受损程度直接进行分级，但肠镜下分级与IT-aGVHD患者预后相关性的研究较少。本研究通过回顾性收集UCBT后出现IT-aGVHD并接受肠镜检查患者的临床资料，比较肠镜下不同受损级别患者aGVHD治疗的有效性及预后，探讨肠镜下分级预测UCBT后IT-aGVHD患者预后的价值。

## 病例与方法

1. 病例：纳入2016年6月至2023年6月在安徽省立医院首次接受UCBT后发生激素耐药的IT-aGVHD的恶性血液病患者50例，其中急性髓系白血病（AML）20例，急性淋巴细胞白血病（ALL）23例，骨髓增生异常综合征（MDS）7例，均接受结肠镜检查。该研究经安徽省立医院伦理委员会批准（批件号：2023-RE-330）。

2. 定义及评价标准：aGVHD分级采用改良的Glucksberg分级标准[11]–[12]，基于各器官系统受累程度的临床分级系统，胃肠道1级指腹泻量为500～1 000 ml/d，2级指腹泻量为1 000～1 500 ml/d，3级指腹泻量为1 500～2 000 ml/d，4级指腹泻量2 000 ml/d以上或伴肠梗阻和剧烈腹痛；1级为轻度，2～4级为重度。aGVHD的疗效评估标准根据《中国异基因造血干细胞移植治疗血液系统疾病专家共识（Ⅲ）——急性移植物抗宿主病（2020年版）》[13]，完全缓解（CR）和部分缓解（PR）为治疗有效。激素耐药aGVHD的判断标准采用2018年欧洲骨髓移植学会-美国国立卫生研究院-国际骨髓移植研究中心的标准，即一线糖皮质激素开始治疗后3～5 d内疗效评估为进展或治疗5～7 d内疗效评估为无反应或包括糖皮质激素在内的免疫抑制剂治疗28 d未达CR。肠镜黏膜受损的分级依据肠镜下黏膜表现，由2位内镜检查医师在检查完成时共同评估，采用Washington等提出的肠镜组织Freiburg分级标准（Ⅰ级的特征性改变：龟纹样改变；Ⅱ级的特征性改变：红斑病灶；Ⅲ级的特征性改变：口疮样溃疡；Ⅳ级的特征性改变：黏膜剥脱伴大片状融合溃疡）[14]–[15]。其中肠镜下黏膜损伤较轻者（Freiburg分级标准Ⅰ、Ⅱ级）为轻症组，肠镜下黏膜损伤较重者（Freiburg分级标准Ⅲ、Ⅳ级）为重症组。巨细胞病毒（CMV）血症定义为UCBT后患者通过实时定量聚合酶链反应曾检测出外周血CMV大于1 000拷贝/ml或经组织病理学检查证实存在CMV感染。对于肝脏aGVHD分级，主要以临床生化指标进行评估，肝脏Ⅰ级指总胆红素<34.2 µmol/L（2 mg/dl），Ⅱ级指总胆红素≥34.2 µmol/L（2 mg/dl）[16]。

3. 移植预处理及aGVHD的防治方案：50例患者均采用不含抗胸腺细胞免疫球蛋白清髓性预处理方案，其中7例以放疗为基础，43例以化疗为基础。所有患者均采用环孢素A联合霉酚酸酯预防aGVHD，IT-aGVHD的一线治疗均使用甲泼尼龙（1～2 mg·kg^−1^·d^−1^），一线治疗无效的患者采用二线治疗，二线治疗包括巴利昔单抗、芦可替尼等。

4. 随访：通过查阅门诊、住院病历及电话随访的方式获取患者移植后生存情况。随访截止日期为2023年11月30日。中位随访时间730（61～730）d。

5. 统计学处理：采用SPSS 24.0和Graphpad Prism 9.0软件进行统计学分析。分类资料采用例数（百分比）表示，定量资料采用中位数（范围）描述，分类变量的组间比较采用卡方检验或Fisher精确检验。采用Kaplan-Meier法进行生存分析，组间生存率的比较采用Log-rank检验。多因素分析采用Logistic回归模型及Cox风险比例回归模型。因总体例数较少，本研究仅纳入既往文献提示对疗效和预后有较大影响的变量进行多因素分析，包括患者的性别、年龄、肠镜下分级、CMV感染状态、原发病种类、肝脏aGVHD分级、脐血回输的CD34^+^细胞数量、中性粒细胞恢复时间、移植前疾病状态、二线治疗方案、首次发生aGVHD的时间及启动二线治疗的时间[4],[6],[12]。*P*<0.05为差异有统计学意义。

## 结果

1. 临床基线特征：本研究共纳入50例UCBT后发生aGVHD、一线治疗效果不佳后接受肠镜检查的恶性血液病患者，其中男26例，女24例，中位年龄20.5（5～70）岁。二线治疗方案包括巴利昔单抗37例（74.0％），巴利昔单抗联合芦可替尼13例（26.0％）。所有患者中轻症组27例（54.0％），重症组23例（46.0％）。37例（74.0％）患者进行了肠道组织活检，病理证实轻症组、重症组分别有7例（18.9％）、10例（27.0％）患者肠道组织存在CMV包涵体，两组的差异无统计学意义（*P*>0.05）。轻症组、重症组男性患者均为13例，年龄≥14岁患者分别为19例、16例。轻症组、重症组分别有13例、10例患者诊断为ALL，11例、9例诊断为AML，3例、4例诊断为MDS。轻症组、重症组预处理方案以氟达拉滨为基础的化疗患者分别为5例、2例，以全身照射为基础的放疗患者分别为22例、21例。轻症组、重症组肠镜检查时IT-aGVHD临床分级轻度患者分别为5例、6例，临床分级重度患者分别为22例、17例。轻症组、重症组分别有19例、18例患者二线治疗采用巴利昔单抗，8例、5例患者二线治疗采用巴利昔单抗+芦可替尼。轻症组、重症组上述临床特征的差异均无统计学意义（*P*均>0.05）。轻症组、重症组分别有6例、16例患者具有CMV血症，差异有统计学意义（*P*＝0.001）。

2. IT-aGVHD对二线治疗的反应：移植后接受肠镜检查的中位时间为63.5（31～287）d，其中轻症组为移植后59（31～247）d，重症组为移植后70（45～287）d，两组的差异无统计学意义（*P*>0.05）。所有患者在肠镜检查前均已接受aGVHD的二线治疗，肠镜检查时接受二线治疗的中位时间为39（0～268）d，其中轻症组为32（1～213）d，重症组39（0～268）d，两组相比差异无统计学意义（*P*>0.05）。进行肠镜检查后的28 d内，轻症组9例（33.3％）患者达到CR，16例（59.3％）达到PR，有效率为92.6％；重症组3例（13.0％）患者达到CR，8例（34.8％）达到PR，有效率为47.8％，轻症组肠镜检查后28 d内aGVHD二线治疗有效率明显高于重症组（*P*<0.001）。进行肠镜检查后的56 d内，轻症组16例（59.3％）患者达到CR，6例（22.2％）达到PR，有效率为81.5％；重症组2例（8.7％）患者达到CR，7例（30.4％）达到PR，有效率为39.1％，轻症组肠镜检查后56 d内aGVHD二线治疗有效率明显高于重症组（*P*＝0.002）。截至末次随访，轻症组17例（63.0％）患者达到CR，8例（29.6％）达到PR，有效率为92.6％；重症组7例（30.4％）患者达到CR，8例（34.8％）达到PR，有效率为65.2％，轻症组肠镜检查后aGVHD二线治疗的最佳有效率明显高于重症组（*P*＝0.040）。多因素分析显示，肠镜下黏膜损伤较重（Freiburg分级标准Ⅲ、Ⅳ级）是影响IT-aGVHD治疗有效率的独立危险因素（*HR*＝19.32，95％*CI* 1.67～223.79，*P*＝0.018）（表1）。

**表1:** 影响肠道aGVHD患者二线治疗疗效的多因素分析

变量	*HR*（95%*CI*）	*P*值
肠镜下黏膜受损程度（重症组，轻症组）	19.32（1.67～223.79）	0.018
性别（男，女）	3.83（0.56～26.27）	0.172
是否出现CMV血症（是，否）	1.10（0.16～7.63）	0.920
肝脏aGVHD分级^a^（Ⅱ级，Ⅰ级）	0.25（0.03～2.19）	0.209
原发病（AML或MDS，ALL）	0.89（0.14～5.59）	0.898
年龄（≥14岁，<14岁）	1.65（0.15～18.69）	0.685
脐血回输的CD34^+^细胞数（>2.415×10^6^/L，≤2.415×10^6^/L）	0.34（0.06～2.04）	0.240
二线治疗方案（巴利昔单抗+芦可替尼，巴利昔单抗）	1.96（0.25～15.17）	0.519
首次发生aGVHD的时间（移植后>23 d，移植后≤23 d）	0.26（0.03～2.28）	0.224
启动二线治疗的时间（移植后>39 d，移植后≤39 d）	1.01（0.99～1.02）	0.347

**注** ^a^临床分级采用改良的Glucksberg分级标准[11]–[12]；aGVHD：急性移植物抗宿主病；CMV：巨细胞病毒；AML：急性髓系白血病；MDS：骨髓增生异常综合征；ALL：急性淋巴细胞白血病

3. 患者生存情况：接受肠镜检查后2年的总生存（OS）率为54.0％（95％*CI* 39.5％～68.5％）。轻症组、重症组患者的2年OS率分别为70.4％（95％*CI* 52.0％～88.8％）和34.8％（95％*CI* 14.8％～54.8％），差异有统计学意义（*P*＝0.003）（图1）。多因素分析显示，肠镜下分级（*HR*＝3.46，95％*CI* 1.05～11.40，*P*＝0.042）、是否出现CMV血症（*HR*＝5.82，95％*CI* 1.88～17.96，*P*＝0.002）、二线治疗方案（*HR*＝0.27，95％*CI* 0.09～0.82，*P*＝0.021）及年龄（*HR*＝10.44，95％*CI* 2.30～47.27，*P*＝0.002）是影响OS的独立危险因素（表2）。截至末次随访，23例患者死亡，其中死于感染12例（52.2％），aGVHD 10例（43.5％），复发1例（4.3％）。

**图1:**
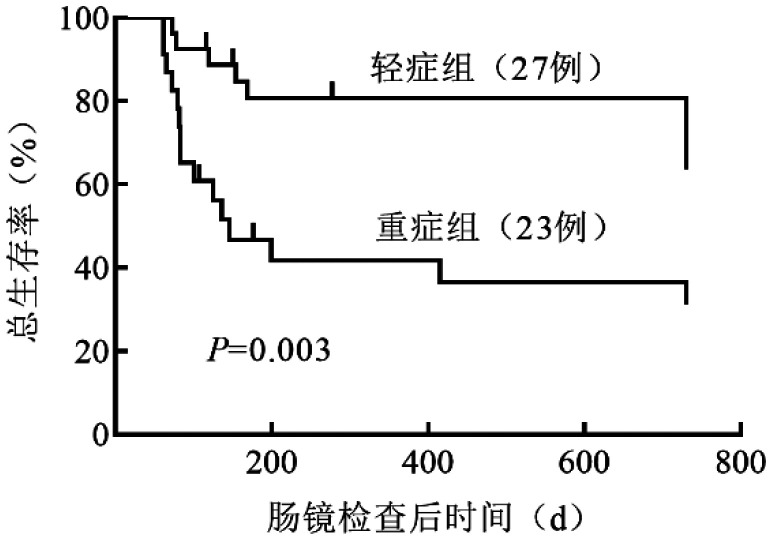
轻症组和重症组患者接受肠镜检查后的总生存曲线

**表2:** 影响肠道aGVHD患者肠镜检查后2年总生存的多因素分析

变量	*HR*（95%*CI*）	*P*值
肠镜下黏膜受损程度（重症组，轻症组）	3.46（1.05～11.40）	0.042
性别（男，女）	1.85（0.75～4.56）	0.180
是否出现CMV血症（是，否）	5.82（1.88～17.96）	0.002
肝脏aGVHD分级^a^（Ⅱ级，Ⅰ级）	1.16（0.41～3.27）	0.777
原发病（AML或MDS，ALL）	1.15（0.46～2.95）	0.778
年龄（≥14岁，<14岁）	10.44（2.30～47.27）	0.002
脐血回输的CD34^+^细胞数（>2.415×10^6^/L，≤2.415×10^6^/L）	1.19（0.45～3.14）	0.731
中性粒细胞恢复时间（移植后>19.5 d，移植后≤19.5 d）	0.66（0.24～1.82）	0.421
移植前疾病状态（未缓解，缓解）	2.15（0.55～8.47）	0.273
二线治疗方案（巴利昔单抗+芦可替尼，巴利昔单抗）	0.27（0.09～0.82）	0.021

**注** ^a^临床分级采用改良的Glucksberg分级标准[11]–[12]；aGVHD：急性移植物抗宿主病；CMV：巨细胞病毒；AML：急性髓系白血病；MDS：骨髓增生异常综合征；ALL：急性淋巴细胞白血病

## 讨论

aGVHD是影响allo-HSCT疗效的重要因素，对一线治疗有效率低等因素导致IT-aGVHD成为移植后患者死亡的主要aGVHD类型[17]。因缺乏可靠的诊断及预后评估指标，临床中仍主要依据临床表现对IT-aGVHD患者进行分层管理。但目前临床广泛使用的标准仍无法准确评估一线治疗失败的IT-aGVHD对二线治疗的有效率及患者的预后。肠镜是诊治肠道疾病的重要手段，因耐受性好、安全性高等特点适用于IT-aGVHD的诊断和鉴别诊断。肠镜虽然可提供活检进行组织病理学检查，但因IT-aGVHD患者多伴严重的血小板减少及明显的出血倾向，部分患者无法安全进行肠镜下活检。美国胃肠镜学会建议，对于PLT>20×10^9^/L的患者可进行诊断性内镜检查，但需要进行标准钳活检术时PLT需维持在50×10^9^/L以上[18]。Oh等[19]也证实当PLT<50×10^9^/L时内镜活检出血风险显著升高。本研究纳入的50例患者在进行肠镜检查时的中位PLT为35×10^9^/L，低于美国胃肠镜学会推荐的PLT水平，导致28例（56％）患者无法进行标准钳活检术。肠镜是一种快速且可重复进行的检查手段，本研究结果显示，肠镜下黏膜受损的严重程度与IT-aGVHD对二线治疗的有效率及患者的预后相关，为IT-aGVHD治疗策略的制定提供了更加可靠的依据。

对于IT-aGVHD，目前主要依靠患者每日腹泻量进行aGVHD严重程度的临床分级，而不同临床分级会采用不同的aGVHD治疗策略[11],[20]–[21]。本研究轻症组患者中有81.5％临床分级为重度，而重症组患者中有26.1％临床分级为轻度，提示肠镜下分级与临床分级存在差异。Oomori等[22]评估了12例接受allo-HSCT后临床诊断为IT-GVHD患者的结肠镜和病理结果，发现内镜检查结果与病理结果均具有诊断意义。而另一项研究同样发现IT-aGVHD患者的内镜检查结果与病理学分级高度相关[23]，提示肠镜下黏膜分级可为IT-aGVHD患者的黏膜受损程度提供更加可靠的信息，较仅依靠患者腹泻量进行临床分级更准确地反映肠道黏膜受损的程度。激素耐药的IT-aGVHD对二线治疗的有效率较差，近期研究显示，糖皮质激素耐药aGVHD的二线治疗有效率为25.0％～72.5％[24]–[27]，目前尚无可靠指标可评估糖皮质激素耐药的IT-aGVHD对二线治疗的有效性。本研究结果显示，肠镜下黏膜损伤的严重程度是影响IT-aGVHD对二线治疗反应的重要因素，黏膜损伤较重患者治疗后的有效率显著低于黏膜损伤较轻的患者。对于临床诊断为轻度IT-aGVHD的患者，若肠镜检查发现黏膜损伤较重，提示该类患者aGVHD治疗的疗效可能较差，需及时调整治疗策略。对于临床诊断为重度IT-aGVHD的患者，若肠镜检查时发现肠道黏膜损伤较轻，提示该类患者对治疗的有效率可能较高，可考虑避免使用强度过高的治疗策略。

病毒性肠炎与肠道GVHD均属于移植后早期严重的肠道并发症，其临床表现与IT-GVHD较难鉴别，内镜及组织病理检查是目前鉴别两者的可靠方法[28]。IT-GVHD和CMV肠炎均有特异性较高的肠镜下黏膜改变，其中，黏膜龟纹样改变和深部溃疡分别是两者较为特征性的改变[29]。有研究表明，GVHD合并CMV会导致患者的治疗时间延长[29]。Yanada等[30]也发现，IT-aGVHD患者合并CMV肠炎会导致患者的治疗更加复杂。本研究的50例患者中有24例为aGVHD合并CMV感染的患者，接受肠镜检查后2年OS率为22.7％，低于其他患者，与既往报道的结果类似，该类患者的预后较单纯的IT-aGVHD患者更差。

本研究也存在一些不足：首先本研究为回顾性研究，患者进行肠镜检查的时间不固定，可能会影响疗效的观察；其次，患者接受的二线治疗不同，主要是由于GVHD的二线治疗目前没有统一的方案；此外，本研究病例数较少，多因素分析纳入的自变量有限，可能遗漏重要的影响因素，同时无法构建更加可靠的临床预测模型。

综上，肠镜下黏膜损伤程度的分级可用于评估激素耐药的IT-aGVHD患者对二线治疗的疗效及患者的预后。对于无法进行活检的患者，肠镜下黏膜损伤较重的患者二线治疗的疗效差，患者的长期生存率较低，对上述患者需制定更有效的aGVHD治疗策略。
